# Harnessing the Biocontrol Potential of *Bradyrhizobium japonicum* FCBP-SB-406 to Manage Charcoal Rot of Soybean with Increased Yield Response for the Development of Sustainable Agriculture

**DOI:** 10.3390/microorganisms12020304

**Published:** 2024-01-31

**Authors:** Umar Khalid, Zill-e-Huma Aftab, Tehmina Anjum, Najat A. Bokhari, Waheed Akram, Waheed Anwar

**Affiliations:** 1 Department of Plant Pathology, Faculty of Agricultural Sciences, University of the Punjab, Lahore 54590, Pakistan; umarkhalid44@gmail.com (U.K.); anjum.dpp@pu.edu.pk (T.A.); waheedanwar.iags@pu.edu.pk (W.A.); 2 Department of Botany and Microbiology, College of Science, King Saud University, Riyadh 11495, Saudi Arabia; najatab@ksu.edu.sa

**Keywords:** biofertilizer, biopesticide, agriculture, disease management

## Abstract

Plant growth-promoting bacteria (PGPRs) have the potential to act as biofertilizers and biopesticides. This study was planned to explore indigenously isolated PGPRs as a potential candidate to control charcoal rot that affects various crops including soybean. Among the four different tested species of PGPRs, *Bradyrhizobium japonicum* (FCBP-SB-406) showed significant potential to enhance growth and control soil borne pathogens such as *Macrophomina phaseolina. Bacillus subtilis* (FCBP-SB-324) followed next. *Bradyrhizobium japonicum* (FCBP-SB-406) reduced disease severity up to 81.25% in comparison to the control. The strain showed a strong fertilizing effect as a highly significant increase in biomass and other agronomic parameters was recorded in plants grown in its presence. The same was supported by the Pearson’s correlation and principal component analysis. A decrease in disease incidence and severity may be due to the induced resistance imparted by the bacterium. This resulted in significant increments in quantities of defense enzymes, including catalase, peroxidase (PO), polyphenol oxidase (PPO), phenylalanine ammonia lyase (PAL) and superoxide dismutase (SOD). A significant production of proteases, catalases and hydrogen cyanide by *B. japonicum* (FCBP-SB-406) can also be associated to mycoparasitism. The establishment of PGPRs in treated soils also showed positive effects on soil health. Total metabolite profiling of treated plants in comparison to the control showed the upregulation of many flavonoids, isoflavonoids and amino acids. Many of these compounds have been well reported with antimicrobial activities. *Bradyrhizobium japonicum* (FCBP-SB-406) can be employed for the production of a potential formulation to support sustainable agriculture by reducing the input of synthetic pesticides and fertilizers.

## 1. Introduction

The genus Glycine most likely evolved from a perennial ancestor in Southeast Asia. Modern soybean (*Glycine max* (L.) Merr.) originated through domestication of the wild annual soybean (*Glycine soja* Sieb. and Zucc.) during the Shang dynasty (1766 to 1125 BCE) [1]. Soybean, the oilseed legume crop, accounts for 80% of the area and 68% of the world’s legume produce [2,3]. Soybean diseases are important as they significantly decrease the quantity and quality of grain grown for food, feed and fuel production. Pathogens, like *Colletotrichum dematium* var. *truncatum*, *Diaporthe phaseolorum*, *Fusarium oxysporum* and *Phytophthora megasperma,* reduce soybean quality, leading to more than 30% yield losses [4]. *Macrophomina phaseolina* is responsible for charcoal rot of soybean and is the most critical monotypic soil-borne fungus that belongs to the class Deuteromycetes and is reported to induce a loss of more than 50% globally [5]. It is a widespread devastating pathogen, which infects the plant within 2 to 3 weeks after sowing when the soils are wet and the temperature is between 28 and 33 °C [6]. Infected plants have small, rolled and yellow leaves, which later wilt, die and remain attached to the petiole. Diseased plants are likely to mature early, with the upper third of the plant having unfilled pods and tiny, black microsclerotia that resemble charcoal powder developing beneath the epidermis on the lower stem, taproot and pith. Black streaks may develop in the woody portion of the root crown, and lower stems may appear silvery or light grey and have black, dusty microsclerotia (survival structures) on the stem surface [7].

Management options for *M. phaseolina* continue to be difficult to implement, despite several research efforts aimed at controlling the disease [8]. Interactions between the pathogen, the host plant and both the abiotic and biotic components of the environment lead to the diseases produced by this soil pathogen [9]. Among the novel alternatives for disease management approaches is the combination of biocontrol agents with some chemical inducers to achieve better control of plant pathogens. Plant growth-promoting rhizobacteria (PGPRs) act as biocontrol agents through the production of siderophores, antibiotics and hydrogen cyanide and inhibit fungal growth through the synthesis of fungal cell wall lysing enzymes and also decrease the shelf life of soil-borne pathogens [10]. Ref. [11] identified an additive effect on induced resistance against bacterial spots in pepper, after a combination treatment composed of a bacterial strain *Bacillus pumilus* INR7 with a chemical inducer benzothiadiazole (BTH) in the field. Ref. [12] found that the combination of different *Bacillus subtilis* strains with acibenzolar-S-methyl increased its suppression capacity against *Fusarium* crown and root rot on tomato plants. This systemically induced resistance enhances the host defense mechanism by activating defense-related proteins, i.e., increased peroxidase (PO) activity, hydrolytic enzymes such as chitinases and proteases that can be harnessed for the control of phytopathogens, along with important plant growth-promoting characteristics, such as mineral phosphate solubilization, siderophore and auxin production [13,14]. With this in view, this study was planned to control charcoal rot in soybean through PGPR’s induced resistance.

## 2. Materials and Methods

### 2.1. Procurement of Cultures of Macrophomina phaseolina and PGPRs

The identified culture of *Macrophomina phaseolina* was procured from the Crop Disease Research Institute (CDRI), NARC, Islamabad, Pakistan. Four antagonistic bacterial cultures, including *Bacillus subtilis* (FCBP-SB-324), *Bradyrhizobium japonicum* (FCBP-SB-406)*, Azospirillum brasilense* (FCBP-SB-25) and *Pantoea agglomerans* (FCBP-PB-454), were obtained from First Fungal Culture Bank of Pakistan (FCBP), Department of Plant Pathology, Faculty of Agricultural Sciences, University of the Punjab, Lahore, Pakistan.

### 2.2. Metabolites Quantification of Selected PGPRs

Protease, catalase and hydrogen cyanide (HCN), which are PGPR metabolites, were measured by growing them in an appropriate medium, as stated below:

#### 2.2.1. Protease Activity

Petri plates with 1% skim milk agar medium were spot inoculated with each selected bacterial strain. After 48 h of incubation at 37 °C, a clear zone around inoculation was observed. A clear zone of up to 5, 10 and 15 mm was taken as low, good and exceptionally good proteolytic activity.

#### 2.2.2. Catalase Activity

A 12 h old culture of each bacterium was mixed with 3% hydrogen peroxide on a glass slide with the help of a sterilized loop. The formation of bubbles was taken as a positive result [15].

#### 2.2.3. Hydrogen Cyanide (HCN) Production

Nutrient agar medium supplemented with 0.44% glycine was streaked with a 24 h old culture. Whatman No. 1 filter paper was then coated with this culture and dipped in a solution of 2% sodium carbonate and 0.5% picric acid. The setup was kept at 30 °C for 72 h. Change in color of the filter paper ranging from yellow to orange, red and brown was taken as low, good and exceptionally good production of HCN.

### 2.3. In Vitro Screening of PGPRs for Antifungal Activity

The dual culture screening technique using potato dextrose agar media was employed to assess the antifungal activity of selected PGPRs. A full bacterial loop was streaked at one side of the Petri plate. After incubating the plates for 2 days, mycelial agar discs of 5 mm were taken from a one-week-old culture and placed on other side of the Petri plate almost 3 cm distant from the bacterial streak. Culture in paired form was then incubated at 27 ± 1 °C. Petri plates inoculated with *M. phaseolina* served as a control. Percentage growth inhibition was quantified by using the formula of [16]. Treatment consisted of five replicate plates, and the experiment was performed twice.

### 2.4. Field Trial to Evaluate Disease Suppression Potential of Selected PGPRs

#### 2.4.1. Preparation of PGPR Inoculum

The competing bacteria were grown in nutrient broth at 28 °C for 24 h at 120 rpm in a shaker incubator. Following the centrifugation of bacterial cultures at 6000 rpm for 15 min, they were resuspended in phosphate buffer (pH 7.4). Optical density (OD) of the bacterial pellet was calibrated to 1 at 600 nm and the supernatant was removed. The bacterial pellet was mixed with distilled water. Seeds were surface sterilized with 0.1% HgCl_2_ and then sequentially rinsed with double-distilled water. Soybean seeds were immersed in a bacterial inoculum for 3–4 h. Seeds were soaked in water that was distilled and autoclaved as a control.

#### 2.4.2. Disease Induction

Sorghum seeds (140 g) were autoclaved for 20 min at 121 °C and 15 psi pressure after being immersed in distilled water overnight. Properly sterilized sorghum seeds were combined with fresh *M. phaseolina* culture, which contained 100 microsclerotia per gram dry weight of soil. The mixture was then incubated for 14 days at 30 °C. The soil was then blended with these infected sorghum seeds, four hours before planting soybean seedlings. Using the dilution plate approach, the frequency of *M. phaseolina* was estimated to be 1.2 × 10^7^ colony-forming units per gram (CFU g^−1^) of sorghum [17].

#### 2.4.3. Field Experiment

The effectiveness of *M. phaseolina* over charcoal rot disease of soybean was evaluated at the experimental station of the Faculty of Agricultural Sciences University of Punjab, Lahore. Two successive field experiments were conducted during the soybean growing seasons of 2022 and 2023. Disease-free seeds of susceptible soybean variety (Ajmeri) were taken from the Nuclear Institute for Agriculture and Biology (NIAB), Faisalabad, and sown on raised beds. The experiment was set up in a fully randomized block design with the field divided into tiny plots (3 × 3 m) that each had two raised beds. The treatments were prepared and used according to the standards of the greenhouse experiment. Each of the four bacterial suspensions (1.0 × 10^6^ CFU/mL) and *M. phaseolina* (inoculum spore suspension at a concentration of 2 × 10^4^ conidia/mL) were treated at a rate of about 350 L ha^−1^. Distilled sterile water was sprinkled over the control plants. No fewer than fifty plants were present in each of the five replicate plots where each treatment was being used. After 10 days of pathogen administration, disease incidence and disease severity were measured as described in Section 2.4.5. Agronomical traits of treated plants (10 Replicates) were also measured, including fresh and dry weight, root and shoot length, root nodule number and number of leaves. Weighing the freshly collected plant sample provided fresh weight. To estimate dry weight, plant samples were dried in an oven for 72 h at 60 °C.

#### 2.4.4. Measurement of Photosynthetic Pigments

Fully grown mature leaves were used to measure the amount of chlorophyll. One gram of fresh leaves was ground with 20–40 mL of 80% acetone. The mixture was then centrifuged at 10,000 rpm for 5 min. The supernatant was transferred, and the procedure was repeated until the residue became colorless. The absorbance of the solution was taken at 646, 663 and 470 nm against the solvent (acetone) blank. Following equations were used to calculate chlorophyll a, b, total chlorophyll and carotenoids [18].

Total Chlorophyll: 18.71(A_646_) + 7.15(A_663_)

Chlorophyll *a*: 12.25(A_663_) − 2.79(A_646_)

Chlorophyll *b*: 21.5(A_646_) − 5.10(A_663_)

Carotenoids = (1000 × A_470_ − 1.82 × Chl *a* − 85.02 × Chl *b*)/198

#### 2.4.5. Calculation of Disease Incidence and Disease Severity

Disease incidence was calculated using the following formula:

Disease incidence: No. of infected plants × 100/Total no. of plant assessed

The [18] algorithm was used to measure the disease severity index. Disease severity was based on a scale from 0 to 11 points, with 0 denoting no symptoms, 1 denoting 1 to 3 percent, 2 denoting 4 to 6 percent, 3 denoting 7 to 12 percent, 4 denoting 13 to 25 percent, 5 denoting 25 to 50 percent, 6 denoting 50 to 75 percent, 7 denoting 75 to 87 percent, 8 denoting 87 to 94 percent, 09 denoting 94 to 97 percent and 10 denoting 97–100 percent [19]. Following formula was used to calculate percentage disease severity index.

$$DSI\left(\%\right)=\frac{\sum \left(Class\;frequency\times score\;of\;rating\;class\right)x^{2}}{\left(total\;number\;of\;observations\right)\times \left(maximal\;disease\;index\right)}\times 100$$

### 2.5. Evaluation of Defense-Related Enzymes

#### 2.5.1. Peroxidase Activity

A 0.5 g sample of fresh leaves was homogenized in 5 mL of 50 mM sodium phosphate buffer with pH 6.8 in a chilled pestle and mortar; this was accompanied by centrifugation of homogenate at 10,000 rpm for 20 min at 4 °C. A reaction mixture containing 1.5 mL of 0.05 M pyrogallol and 0.5 mL of 1% H_2_O_2_ was added in 0.5 mL of enzyme extract. The OD was taken at 420 nm. The enzyme units were calculated in µmol min^−1^ mg^−1^.

#### 2.5.2. Polyphenol Oxidase Activity

In a chilled pestle and mortar, 0.5 g of leaves was crushed with 5 mL of 0.1 M phosphate buffer (pH 6.5). The mixture was centrifuged at 10,000 rpm for 15 min at 4 °C to obtain the supernatant containing enzyme extract. The reaction mixture contained 1.5 mL of sodium phosphate buffer (pH 6.5) along with 200 µL of enzyme extract, and 200 µL of freshly prepared 0.5 M catechol was added; absorbance was measured at 420 nm. The enzyme units were calculated in µmol min^−1^ mg^−1^.

#### 2.5.3. Phenylalanine Ammonia Lyase Activity

In a chilled pestle and mortar, 0.5 g of leaves was crushed with 25 mM Tris HCl buffer (pH 8.8). The mixture was centrifuged at 10,000 rpm for 15 min at 4 °C to obtain the supernatant. The reaction mixture of 1 mL (0.1 mL enzyme extract + 0.5 mL 50 mM L-phenylalanine + 0.4 mL Tris HCl buffer) was incubated for 2 h at 40 °C, followed by the addition of 60 µL of 5 N HCl to stop the reaction. Same volume of reaction mixture without L-phenylalanine was taken as control. The OD was taken at 290 nm. The enzyme units were calculated in µmol min^−1^ mg^−1^.

#### 2.5.4. Superoxide Dismutase Activity

Crushed leaves (0.5 g) in buffer were centrifuged at 10,000 rpm for 20 min at 4 °C to obtain the crude enzyme extract. Further, 3 mL of reaction mixture containing 50 mM sodium phosphate buffer (pH 7.6), 50 mM sodium carbonate, 50 µM NBT, 0.1 mM EDTA, 10 µM riboflavin, 12 mM L-methionine and 100 µL of crude extract was exposed to white light for 15 min at room temperature. The absorbance was taken at 560 nm. The reaction mixture for control was without any crude extract. The enzyme units were calculated in µmol min^−1^ mg^−1^.

#### 2.5.5. Catalase Activity

Fresh leaves (0.5 g) were homogenized in 2 mL of 50 mM potassium phosphate buffer with pH 7.0. The resultant lysate was centrifuged for 10 min at 3000 rpm. A water bath was used to incubate the reaction mixture (0.2 mL enzyme extract + 5 mM potassium phosphate buffer (pH 7.0), + 0.3 mL of 0.1 M H_2_O_2_) for 30 min. By measuring the decline in absorbance at 240 nm caused by the consumption of H_2_O_2_, enzyme activity was observed. The enzyme units were calculated in µmol min^−1^ mg^−1^.

### 2.6. Physico-Chemical Characterization of Field Soil

Further, 20 g of each soil sample was mixed with 50 mL of deionized water in a shaker for 30 min. The mixture was then filtered through a filter paper. The filtrate was used to measure pH, total dissolved solids and electrical conductivity (EC) by a pH meter and EC meter.

To measure the organic matter, each soil sample in 10 g was taken in porcelain crucible and placed inside a Muffle furnace at 500 °C for 24 h. The quantity measured was based on weight loss upon ignition [20]. Field capacity in percentage was calculated by subtracting the dry weight of the soil from the weight of the soil at field capacity.

### 2.7. LC-Ms Profiling of Total Metabolites of Treated and Un-Treated Plants

A UHPLC-ESI-QQQ-MS/MS analysis of total metabolite of control and soybean plants treated with *B. japonicum*-406 was conducted. Leaves obtained from the soybean plants were ground to fine powder in the pestle and mortar using liquid nitrogen. The extraction was performed using a mixture of methanol and water (80/20, *v*/*v*) containing 1 ng/μL of the internal standard. The obtained extract was centrifuged to settle the debris and passed by a microfilter assembly. Chromatographic separation was performed on an Agilent 1200 ultra-performance liquid chromatography system (Agilent, Santa Clara, CA, USA) with a C18 analytical column (Agilent) and coupled with a Triple-Quad tandem mass spectrometer (6470) system. The mobile phase “A” consisted of 0.1% formic acid (*v*/*v*) in deionized water, whereas the mobile phase B consisted of 0.1% formic acid (*v*/*v*) in methanol. The chromatographic conditions were 95% A and 5% B for the first 5 min, solvent A decreased to 45% and B increased to 55% up to 22 min, solvent A 5% and B 95% over the course of 3 min and remained unchanged for one minute, solvent A 95% and B 5% for 3 min until the end of the run. The MS scan range was 50 to 1500 *m*/*z*. The acquired data were analyzed by MzMine 2.53 software. Compounds were identified using the NIST MS/MS library operated by the MS search program coupled with the MzMine 3 software.

### 2.8. Statistical Analysis

The results of experiments were subjected to analysis of variance (ANOVA) using Tukey’s test using computer-aided software DSAASTAT 1.101. Treatments of means were compared using least significance test (LSD) at *p* < 0.5. Principal component analysis correlation was applied to mean values of all variables using Origin 22. The experiments were run in triplicate.

## 3. Results

### 3.1. Biochemical and Antifungal Activity of Selected PGPRs

Selected bacterial strains were checked for catalase and protease activity and HCN production (Table 1). *Bradyrhizobium japonicum*-406 was found with the highest catalase activity, whereas *B. japonicum*-406, *Bacillus subtilis*-324 and *Pantoea agglomerans*-454 showed equal potential for protease activity and HCN production. However, *Azospirillum brasilense*-25 showed the lowest activity for all three metabolites. When these bacterial strains were checked for their antifungal potential against *Macrophomina phaseolina, B. japonicum*-406 showed the highest activity of 62%, followed by 55% growth reduction by *B. subtilis*-324 and *Pantoea agglomerans*-454 by 49%. The lowest antifungal activity was recorded in the case of *A. brasilense*-25 (Table 1).

### 3.2. Field Experiment

#### 3.2.1. Plant Disease Incidence

**Figure 1:**
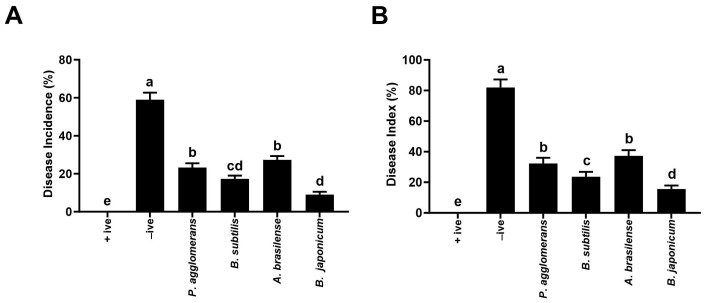
Effect of PGPRs on disease incidence (**A**) and disease index (**B**) of charcoal rot of soybean. −ive (uninfected uninoculated control), +ive (soil infested with *M. phaseolina*). Vertical bars represent standard error between replicates of the same treatment. Small letters show level of significance as governed by ANOVA and Tukey’s multiple range test at *p* < 0.05.

Overall, all the selected PGPRs significantly reduced disease incidence and severity when compared to the positive control (Figure 1). However, the highest reduction in disease index and severity was recorded in plots that received *Bradyrhizobium japonicum*-406 along with the pathogen. The treated plants showed a 66.5% lower disease index and 81.25% lower disease severity in comparison to plants that were grown only in the presence of *Macrophomina phaseolina*. This was followed by the *Bacillus subtilis*-324 as plants grown in its presence showed a 55.4% lower disease index, as compared to the control. *Azospirillum brasilense*-434 and *Pantoe agglomerans*-454 were found to be equally significant in reducing charcoal rot. Although the effect was less than *B. japonicum* and *B. subtilis,* both the bacterial species significantly reduced disease incidence up to 31–37%.

#### 3.2.2. Agronomic Parameters and Photosynthetic Pigments

The treatments positively affected various agronomic parameters (Table 2), and the tested PGPRs followed the same pattern as observed in their efficacy of reducing disease incidence. An increase of up to 24.36% in shoot and root length was recorded in plants grown in the presence of *B. japonicum*-406 and *Bacillus subtilis*-324 but in the absence of the selected pathogen. In the presence of the pathogen, the increase in both above- and underground portions was up to 5% more than the negative control. The highest root nodule formation was recorded in plants grown in soils added with *B. japonicum*-406. In comparison to the negative control, an increase of 27% in root nodulation was recorded in plants grown in soils with *B. japonicum*-406, whereas a 6.8% increment was shown by plants grown in the presence of *B. japonicum*-406 and *M. phaseolina.* Significant increments were also recorded in the fresh and dry weights of both aerial and underground plant parts.

**Figure 2:**
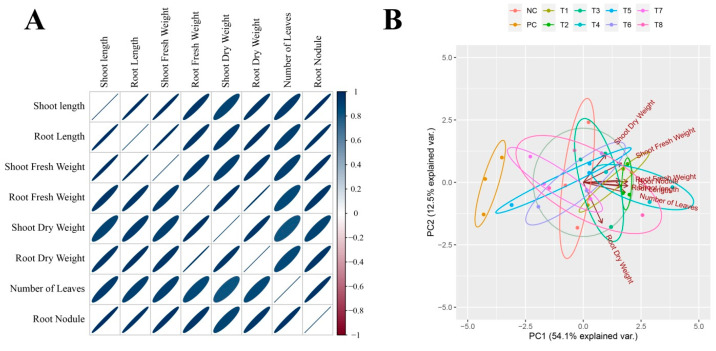
Pearson’s correlation and principal component analysis of the plant samples. (**A**) Correlation pattern (Pearson) of plant growth-related traits. (**B**) Principal component analysis (PCA) plotted from different treatment groups.

A correlation matrix and principal component analysis were used to analyze the complex interplay of growth parameters in plants. Biomass accumulation exhibited strong associations with a number of plant leaves. Principal component analysis (PCA) was utilized to provide insights into the distinctive responses of treatments and growth attributes (Figure 2). The evident clustering in the PCA plot highlights the varying responses of plants against different treatments. In addition, the vectors demonstrate a higher correlation for dry biomasses of plants.

This increase in plant length and fresh and dry weights was found correlated with an increase in photosynthetic pigments (Table 3). Chlorophyll “a” showed a more significant increase than chlorophyll “b” and carotenoids. An increase of 81.25% was measured in the total chlorophyll content of plants grown in the presence of *B. japonicum*-406, whereas plants from plots with both *B. japonicum*-406 and the selected pathogen showed an increment of 43.75%. Carotenoids showed comparatively fewer increments. Increases of 63.6 and 36.4% were recorded in carotenoids of plants grown in the presence of *B. japonicum*-406 alone and in the presence of *M. phaseolina.*

#### 3.2.3. Soil Analysis

Soil analysis at the end of the field trial showed a positive effect in all selected PGPRs on various soil parameters (Table 4). Plots that received *Bradyrhizobium japonicum*-406 alone or in combination with the selected pathogen showed the highest significant improvement in soil health. This PGPR had highly significantly increased organic matter in plots, both when used alone and in the presence of *M. phaseolina.* The increase in organic matter reduced soil pH from 7.6 to 6.2 in plots that received no pathogen. Overall, this positively affected the field capacity. Similarly electrical conductivity (EC) and total dissolved solids (TDS) also reduced in treated plots that supported microbial activity.

#### 3.2.4. Analysis of Plant Defense Enzymes

**Figure 3:**
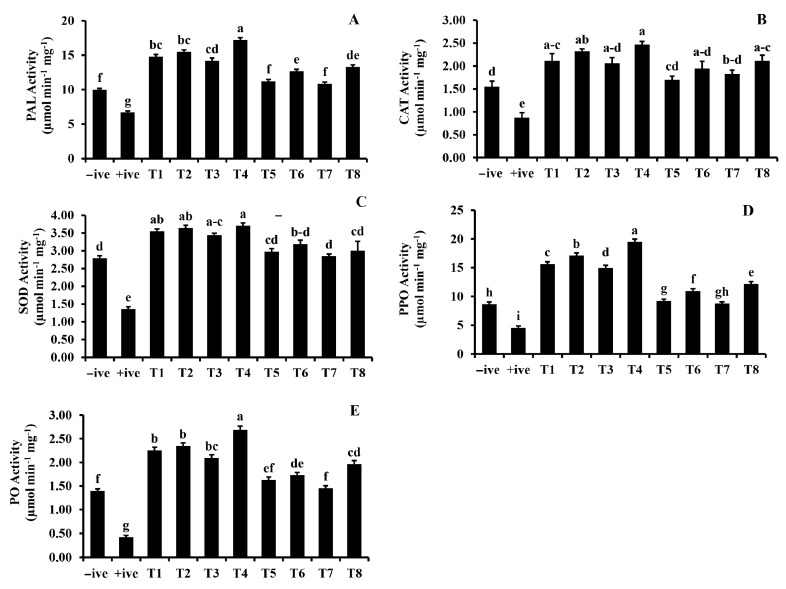
Activities of defense related enzymes (**A**) Phenylammonia lyase; (**B**) catalase; (**C**) super oxide dismutase; (**D**) polyphenol oxidases; and (**E**) peroxidases. −ive (Uninfected uninoculated control), +ive (Soil infested with *M. phaseolina*), T1–T4: soils with *Pantoea agglomerans, Bacillus subtilis, Azospirillum brasilense* and *Bradyrhizobium japonicum* respectively along with *M. phaseolina,* T5–T8: soils only with *Pantoea agglomerans, Bacillus subtilis, Azospirillum brasilense* and *Bradyrhizobium japonicum,* respectively.

Various treatments including the selected PGPRs significantly increased enzyme activity, including catalase, peroxidase (PO), polyphenol oxidase (PPO), phenylalanine ammonia lyase (PAL) and superoxide dismutase (SOD) (Figure 3). Plants grown in the presence of *Bradyrhizobium japonicum*-406 showed the highest increase in all the enzymes, hence confirming the quickest induction of defense response in treated plants. However, the increase in all these defense enzymes was more pronounced in the presence of the pathogen. The highest increment was recorded in PPO, the quantity of which doubled in plants grown in soil inoculated with *B. japonicum*-406 along with the pathogen. This was followed by PO and PAL, which showed an increase of 88.2 and 73%, respectively, in comparison to the negative control. Plants from the same plots showed an increase of 60% in catalase activity, whereas the lowest increment (32.7%) was recorded in SOD activity of this treatment.

### 3.3. Major Compounds Detected in Treated and Control Plants of Soybean through LC/MS

**Figure 4:**
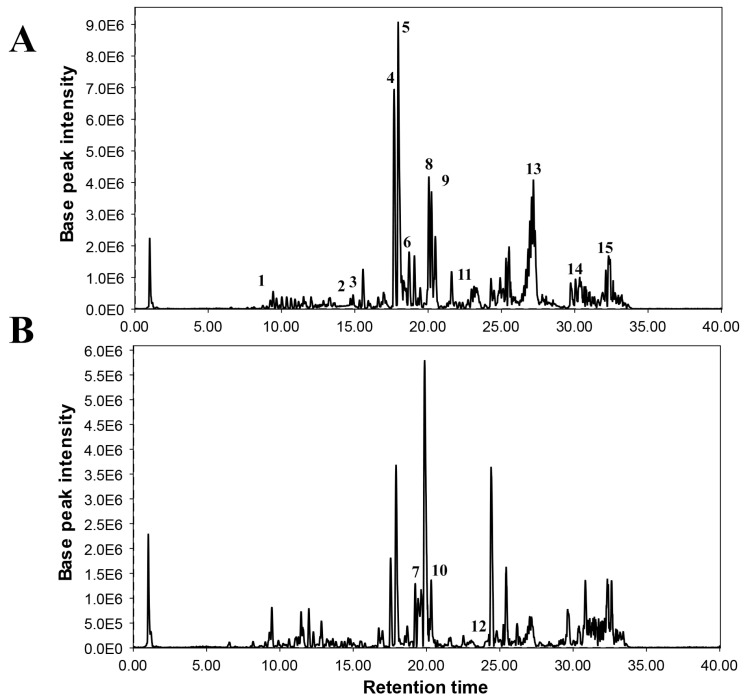
LC/MS based chemical profiling of soybean plants treated with *B. japonicum*-406 (**A**) and control (**B**). Peak 1: Carbamic acid; 2: β-cryptoxanthin; 3: Acetic acid; 4: Prunetin; 5: Tetramethoxy isoflavonone; 6: Glycitin; 7: Quercetin; 8: Malic acid; 9: Octadecanoic acid; 10: Phenyl alanine; 11: Tryptophane; 12: Arginine; 13: Phenylpropanoic acid; 14: Genistein and 15: 7-hydroxy-3-(4-hydroxyphenyl)-4H-chromen-4-one).

Total metabolite profiling of control and soybean plants treated with *B. japonicum*-406 revealed significant differences among various identified phytochemicals (Table 5). Many of the identified compounds were generally found upregulated in plants grown in the presence of *B. japonicum*-406 compared to the control plants (Figure 4). The highest upregulation was recorded in octadecanoic acid that showed a 7.8-fold increase in production of treated plants. Various flavonoids and isoflavones, including prunetin, quercetin, glycitin, tetramethoxy isoflavonone, Genistein and 7-hydroxy-3-(4-hydroxyphenyl)-4H-chromen-4-one, were identified. Among this, the highest increase of almost 7-fold was recorded in Glycitin. Prunetin followed this with a 5-fold increment in treated plants. Quantities of amino acids such as phenyl alanine and tryptophan also doubled. Compounds, like malate, pyruvate, ascorbic acid, malic acid and arginine, showed downregulation in plants treated with *B. japonicum*-406 (Table 5).

## 4. Discussion

Rhizobacteria including *Bradyrhizobium japonicum* are well documented for their crop growth-enhancing potential due to their symbiotic and nitrogen-fixing properties. However, recent studies have also confirmed their role in reducing disease incidence, especially in legume crops [21]. In the present study, four plant growth-promoting bacteria were tested to reduce the charcoal rot incidence on soybean and when compared, *B. japonicum*-406 was found most efficient. *Macrophomina phaseolina* was selected as a test pathogen because no earlier study has been carried out to manage this disease using *B. japonicum* in soybean. Also, *M. phaseolina* is difficult to manage through chemicals due to its persistent and thick-walled seclerotia in soil and the non-affectivity of many systemic and non-systemic fungicides [9]. Field trials showed a significant reduction in disease in plants grown in the presence of *B. japonicum*-406. This potential of the *B. japonicum* can be attributed to mycoparasitism, induced systemic resistance and plant growth promotion potential, as shown in earlier studies [22,23].

The production of metabolites, including HCN, proteases and catalases, is also linked with mycoparasitism. Rhizobacteria are well known for the production of HCN that form complexes with essential elements required for protein functioning and can also disrupt the electron transport chain, causing the death of microorganisms [24]. Among the four tested bacteria, *B. japonicum*-406 showed the highest production of HCN. Similarly, several cell wall hydrolyses are produced by PGPRs that deconstruct the cell walls of the pathogen [25]. *Bradyrhizobium japonicum*-406 tested in this study was found to be an efficient producer of proteases and catalases that can be linked to their biocontrol potential.

Induced systemic resistance is another well-known phenomenon for disease suppression that does not involve the direct inhibition of the pathogen but strengthens physical and chemical barriers of the host, making it strong in fighting the pathogen. Various species of *Bradyrhizobium* have been documented to induce systemic resistance in different crops. However, the mechanism of inducing systemic resistance may vary from species to species. Ref. [26] reported the biocontrol potential of *B. japonicum* BRC 2485 against bacterial wilt pathogen through induced resistance triggered because of the production of abscisic acid. Several other studies have also shown that rhizobacteria have potential to elicit induced resistance in leguminous plants against diverse pathogens [27,28,29,30].

The induction of induced resistance is associated with several cellular defense-related chemical reactions that involve defense enzymes. Several defense-related enzymes, including peroxidases, polyphenol oxidases, phenylalanine ammonia lyases and superoxide dismutease, were found to increase significantly in plants grown in the presence of *B. japonicum*-406 and, hence, can be correlated to the induction of defense in these plants. The highest increase was estimated in polyphenol oxidases, which is known to oxidize phenols to o-quinones, leading to the formation of melanin [31]. This was followed by a significant increment in quantities of peroxidases that works along with polyphenol oxidases and is responsible for lignification, cross-linking of phenolics and glycoproteins, suberization, phytoalexin production and initiation of hypersensitive response [32]. Increment in phenylalanine ammonia lyases and superoxide dismutase was also increased at a significant level, confirming the triggering of the phenyl propanoid pathway, resulting in defense induction.

The colonization of rhizosphere by PGPRs results in improved soil health through diverse mechanisms. These bacteria secrete several organic compounds that lower the pH of the soil [33]. As most of the Pakistani soils have high alkaline pH, this characteristic of PGPRs helps improve plant growth significantly. The decomposition of crop residues in soils by PGPRs also the increases organic matter, and, thus, the water holding capacity of the soil improves. Similar effects were recorded in this study during field the trial.

The whole metabolomics analysis of treated and control soybean plants showed clear differentiation. Most of the identified compounds increased in plants grown in the presence of *B. japonicum*-406. Several folds of increment were recorded in flavonoids, including prunetin, quercetin, glycitin and tetramethoxy isoflavonone. Flavonoids are already known for their antimicrobial activity [34]. Prunetin, an O-methylated isoflavone, quercetin, a flavonoid, and tetra methoxy isoflavone are commonly reported in many plants including soybean and are known to have antimicrobial activities [35,36,37,38]. Glycitin is also a typical isoflavone of soybean with reported antimicrobial and antiviral activities [39].

Amino acids showed variation in their quantities. A few amino acids such as phenyl alanine and tryptophan increased significantly, whereas others including arginine decreased in plants grown in the presence of *B. japonicum*-406. The accumulation of phenylalanine and tryptophan can be taken as a clue of the redirection of primary metabolism towards secondary metabolism to produce defense-related compounds. Ref. [40] also reported an increase in aromatic amino acids in tomato plants primed with growth-promoting bacteria. A lower quantity of arginine in treated plants shows a fast utilization rate of this amino acid. Arginine is known to play a key role in polyamines formation linked to plant defense. Lower quantities of this amino acid can be taken as a reason for its quick utilization in defense metabolic reactions [41].

The quantity of malic acid also reduced in plants grown in the presence of *B. japonicum*-406. The role of malic acid secreted by the roots in attracting beneficial bacteria towards the plant is well reported [42]. A reduced quantity of malic acid in aerial parts of the treated plants in comparison to the control may be due to more exudation in the root region, resulting in its strong chemotaxis properties. Among other acids detected, octadecanoic acid and phenyl propanoic acid increased significantly in the treated plants. Octadecanoic acid is an 18-carbon fatty acid that is commonly known as stearic acid, and plants are known to synthesize many antibiotic compounds through it; hence, it also plays an important role in plant defense [43]. Phenyl propanoic acid was also increased significantly in plants grown in the company of *B. japonicum*-406. Phenylpropanoids are plant metabolites involved in defense against pathogens. Through this, several building blocks are produced, such as flavonoids, isoflavonoids, phenolics, suberin, lignin, coumarins, etc., that are responsible for the provision of mechanical and chemical resistance against pathogens [44]. Hence, *B. japonicum*-406 proved its potential to trigger resistance in treated plants up to the level that significantly reduced disease incidence.

## 5. Conclusions

The concept of sustainable agriculture is based on a reduced use of synthetic fertilizers and pesticides that can be achieved through the use of plant growth-promoting bacteria. In this vein, new potential indigenous candidates must be explored. Among the four tested PGPRs, *Bradyrhizobium japonicum*-406 proved itself as the most potent candidate for a biocontrol formulation. The bacterium not only increased the growth of the tested crop but also reduced charcoal rot incidence and severity. Studies showed that the bacterium induced reduced resistance in soybean against soil-borne pathogens, i.e., *Macrophomina phaseolina.*

## Figures and Tables

**Table 1:** Biochemical and antifungal activity of PGPRs.

Sr. No.	Bacterial Strains	Catalase	HCN	Protease	Antifungal Activity (%)
1.	*Bacillus subtilis*	++	++	++	55
2.	*Bradyrhizobium japonicum*	+++	++	++	62
3.	*Azospirillum brasilense*	+	+	+	34
4.	*Pantoea agglomerans*	++	++	++	49

Where: +: low activity; ++: good activity; +++: exceptionally good activity. Antifungal activity was measured in terms of percentage growth inhibition of *Macrophomina phaseolina*.

**Table 2:** Effects of different PGPR strains on different agronomic parameters of soybean.

Treatments	Shoot Length (cm)	Root Length (cm)	Number of Leaves	Root Nodules	Shoot Fresh Weight (g)	Shoot Dry Weight (g)	Root Fresh Weight (g)	Shoot Fresh Weight (g)
**−ive**	**27.54 ± 0.99 ^d^**	**13.94 ± 0.19 ^cd^**	**27.67 ± 1.37 ^d^**	**44.33 ± 1.03 ^d^**	**3.35 ± 0.02 ^c^**	**0.67 ± 0.005 ^bc^**	**2.42 ± 0.06 ^c–e^**	0.48 ± 0.01 ^c–e^
+ive	18.24 ± 0.42 ^f^	8.69 ± 0.44 ^f^	21.33 ± 1.37 ^e^	32.67 ± 1.37 ^e^	2.20 ± 0.16 ^e^	0.44 ± 0.03 ^e^	1.48 ± 0.07 ^f^	0.30 ± 0.01 ^f^
T1	24.95 ± 0.56 ^e^	13.13 ± 0.29 ^de^	27.33 ± 0.52 ^d^	42.33 ± 0.52 ^d^	3.17 ± 0.02 ^cd^	0.63 ± 0.005 ^cd^	2.35 ± 0.09 ^de^	0.47 ± 0.02 ^de^
T2	27.06 ± 0.44 ^d^	13.49 ± 0.37 ^de^	27.67 ± 1.37 ^d^	44.00 ± 0.89 ^d^	3.21 ± 0.02 ^cd^	0.64 ± 0.005 ^cd^	2.57 ± 0.09 ^b–d^	0.51 ± 0.02 ^b–d^
T3	24.31 ± 0.55 ^e^	12.79 ± 0.29 ^e^	26.33 ± 1.03 ^d^	41.67 ± 1.03 ^d^	3.03 ± 0.07 ^d^	0.61 ± 0.01 ^d^	2.22 ± 0.05 ^e^	0.44 ± 0.01 ^e^
T4	28.37 ± 0.49 ^d^	14.55 ± 0.31 ^c^	28.67 ± 0.52 ^cd^	47.33 ± 0.52 ^c^	3.33 ± 0.04 ^c^	0.67 ± 0.01 ^bc^	2.68 ± 0.16 ^a–c^	0.54 ± 0.03 ^a–c^
T5	32.34 ± 0.3 ^bc^	16.49 ± 0.33 ^a^	31.33 ± 1.37 ^bc^	50.33 ± 1.03 ^b^	3.85 ± 0.04 ^ab^	0.67 ± 0.01b ^bc^	2.68 ± 0.16 ^a–c^	0.54 ± 0.03 ^a–c^
T6	32.92 ± 0.76 ^ab^	16.71 ± 0.41 ^a^	34.33 ± 1.37 ^b^	52.67 ± 1.03 ^b^	3.95 ± 0.04 ^a^	0.69 ± 0.006 ^ab^	2.78 ± 0.07 ^ab^	0.56 ± 0.01 ^ab^
T7	30.74 ± 0.34 ^c^	15.54 ± 0.24 ^b^	28.33 ± 1.37 ^cd^	47.67 ± 0.52 ^c^	3.74 ± 0.04 ^b^	0.66 ± 0.01 ^bc^	2.63 ± 0.07 ^a–d^	0.53 ± 0.01 ^a–d^
T8	34.25 ± 1.05 ^a^	17.13 ± 0.53 ^a^	37.67 ± 1.370 ^a^	56.33 ± 1.37 ^a^	3.98 ± 0.04 ^a^	0.71 ± 0.02 ^a^	2.90 ± 0.13 ^a^	0.58 ± 0.03 ^a^

Where: −ive (Uninfected uninoculated control), +ive (Soil infested with *M. phaseolina*), T1–T4: soils with *Pantoea agglomerans*, *Bacillus subtilis*, *Azospirillum brasilense* and *Bradyrhizobium japonicum* respectively along with *M. phaseolina*, T5–T8: soils only with *Pantoea agglomerans*, *Bacillus subtilis*, *Azospirillum brasilense* and *Bradyrhizobium japonicum* respectively. Data represent the mean ± standard error. Different letters denote statistically significant differences between treatments as evaluated by Tukey’s multiple range test at the *p* < 0.05 level. The data is expressed as the average of three replications and three repetitions.

**Table 3:** Effects of different PGPR strains on photosynthetic pigments of soybean plants.

Treatments	Chlorophyll a (mg/g FW)	Chlorophyll b (mg/g FW)	Total Chl (mg/g FW)	Carotenoids (mg/g FW)
−ive	0.193 ± 0.015 ^c^	0.13 ± 0.012 ^c^	0.32 ± 0.026 ^d^	0.11 ± 0.012 ^cd^
+ive	0.093 ± 0.015 ^d^	0.057 ± 0.007 ^d^	0.15 ± 0.021 ^e^	0.05 ± 0.006 ^e^
T1	0.233 ± 0.02 ^bc^	0.12 ± 0.012 ^c^	0.35 ± 0.032 ^cd^	0.11 ± 0.009 ^cd^
T2	0.277 ± 0.02 ^abc^	0.133 ± 0.009 ^bc^	0.41 ± 0.012 ^bcd^	0.13 ± 0.012 ^bcd^
T3	0.213 ± 0.02 ^c^	0.11 ± 0.012 ^cd^	0.32 ± 0.0032 ^d^	0.10 ± 0.012 ^d^
T4	0.303 ± 0.012 ^ab^	0.153 ± 0.015 ^bc^	0.46 ± 0.026 ^bc^	0.15 ± 0.012 ^abc^
T5	0.307 ± 0.02 ^ab^	0.143 ± 0.009 ^bc^	0.45 ± 0.012 ^bc^	0.13 ± 0.009 ^bcd^
T6	0.316 ± 0.023 ^ab^	0.187 ± 0.012 ^ab^	0.50 ± 0.035 ^ab^	0.16 ± 0.009 ^ab^
T7	0.247 ± 0.02 ^bc^	0.133 ± 0.009 ^bc^	0.38 ± 0.029 ^bcd^	0.12 ± 0.009 ^ab^
T8	0.353 ± 0.02 ^a^	0.227 ± 0.018 ^a^	0.58 ± 0.038 ^a^	0.18 ± 0.012 ^a^

Where: −ive (Uninfected uninoculated control), +ive (Soil infested with *M. phaseolina*), T1–T4: soils with *Pantoea agglomerans, Bacillus subtilis, Azospirillum brasilense* and *Bradyrhizobium japonicum* respectively along with *M. phaseolina,* T5–T8: soils only with *Pantoea agglomerans, Bacillus subtilis, Azospirillum brasilense* and *Bradyrhizobium japonicum* respectively. Data represent the mean ± standard error. Different letters denote statistically significant differences between treatments as evaluated by Tukey’s multiple range test at the *p* < 0.05 level. The data is expressed as the average of three replications and three repetitions.

**Table 4:** Soil conditions at different PGPR treatments with and without pathogen.

Treatments	TDS (mgL^−1^)	EC (dSm^−1^)	Organic Matter (%)	Soil pH	Field Capacity %
−ive	79.13	0.74	0.51	7.6	30.5
+ive	78.65	0.72	0.52	7.5	26.8
T1	73.51	0.41	1.23	6.7	27.7
T2	69.82	0.31	1.34	6.3	28.6
T3	68.04	0.36	1.11	6.1	28.5
T4	66.46	0.27	1.13	6.3	30.6
T5	71.53	0.37	1.17	6.8	28.3
T6	68.27	0.29	1.34	6.3	30.9
T7	68.83	0.32	1.34	6.3	28.5
T8	65.65	0.26	1.39	6.2	31.5

Where: −ive (Uninfected uninoculated control), +ive (Soil infested with *M. phaseolina*), T1–T4: soils with *Pantoea agglomerans, Bacillus subtilis, Azospirillum brasilense* and *Bradyrhizobium japonicum* respectively along with *M. phaseolina,* T5–T8: soils only with *Pantoea agglomerans, Bacillus subtilis, Azospirillum brasilense* and *Bradyrhizobium japonicum* respectively.

**Table 5:** Major compounds detected in treated and control plants of soybean by performing LC/MS.

Retention Time	Compound Name	Retention Time	Compound Name
9.122	Carbamic acid	**18.102**	Tetra methoxy isoflavonone
10.013	Fructose	**18.891**	Glycitin
10.205	Ascorbate	**19.492**	Quercetin
10.417	Glucose-6-phosphate	**20.363**	Malic acid
11.701	Glutamic acid	**20.131**	Octadecanoic acid
12.003	Malate	**21.411**	Phenyl alanine
12.412	Pyruvate	**21.912**	Tryptophan
12.843	Ascorbic acid	**24.624**	Arginine
14.731	β-Cryptoxanthin	**27.479**	Phenylpropanoic acid
14.895	Acetic acid	**30.413**	Genistein
17.907	Prunetin	**32.411**	7-hydroxy-3-(4-hydroxyphenyl)-4H-chromen-4-one)

## Data Availability

Data contained within the article.
